# FtsZ-less prokaryotic cell division as well as FtsZ- and dynamin-less chloroplast and non-photosynthetic plastid division

**DOI:** 10.3389/fpls.2014.00459

**Published:** 2014-09-15

**Authors:** Shin-ya Miyagishima, Mami Nakamura, Akihiro Uzuka, Atsuko Era

**Affiliations:** ^1^Center for Frontier Research, National Institute of GeneticsMishima, Japan; ^2^Department of Genetics, Graduate University for Advanced Studies (SOKENDAI)Mishima, Japan; ^3^Japan Science and Technology Agency, CRESTKawaguchi, Japan

**Keywords:** chloroplast division, plastid division, dynamin, endosymbiosis, FtsZ

## Abstract

The chloroplast division machinery is a mixture of a stromal FtsZ-based complex descended from a cyanobacterial ancestor of chloroplasts and a cytosolic dynamin-related protein (DRP) 5B-based complex derived from the eukaryotic host. Molecular genetic studies have shown that each component of the division machinery is normally essential for normal chloroplast division. However, several exceptions have been found. In the absence of the FtsZ ring, non-photosynthetic plastids are able to proliferate, likely by elongation and budding. Depletion of DRP5B impairs, but does not stop chloroplast division. Chloroplasts in glaucophytes, which possesses a peptidoglycan (PG) layer, divide without DRP5B. Certain parasitic eukaryotes possess non-photosynthetic plastids of secondary endosymbiotic origin, but neither FtsZ nor DRP5B is encoded in their genomes. Elucidation of the FtsZ- and/or DRP5B-less chloroplast division mechanism will lead to a better understanding of the function and evolution of the chloroplast division machinery and the finding of the as-yet-unknown mechanism that is likely involved in chloroplast division. Recent studies have shown that FtsZ was lost from a variety of prokaryotes, many of which lost PG by regressive evolution. In addition, even some of the FtsZ-bearing bacteria are able to divide when FtsZ and PG are depleted experimentally. In some cases, alternative mechanisms for cell division, such as budding by an increase of the cell surface-to-volume ratio, are proposed. Although PG is believed to have been lost from chloroplasts other than in glaucophytes, there is some indirect evidence for the existence of PG in chloroplasts. Such information is also useful for understanding how non-photosynthetic plastids are able to divide in FtsZ-depleted cells and the reason for the retention of FtsZ in chloroplast division. Here we summarize information to facilitate analyses of FtsZ- and/or DRP5B-less chloroplast and non-photosynthetic plastid division.

## Introduction

**Figure 1:**
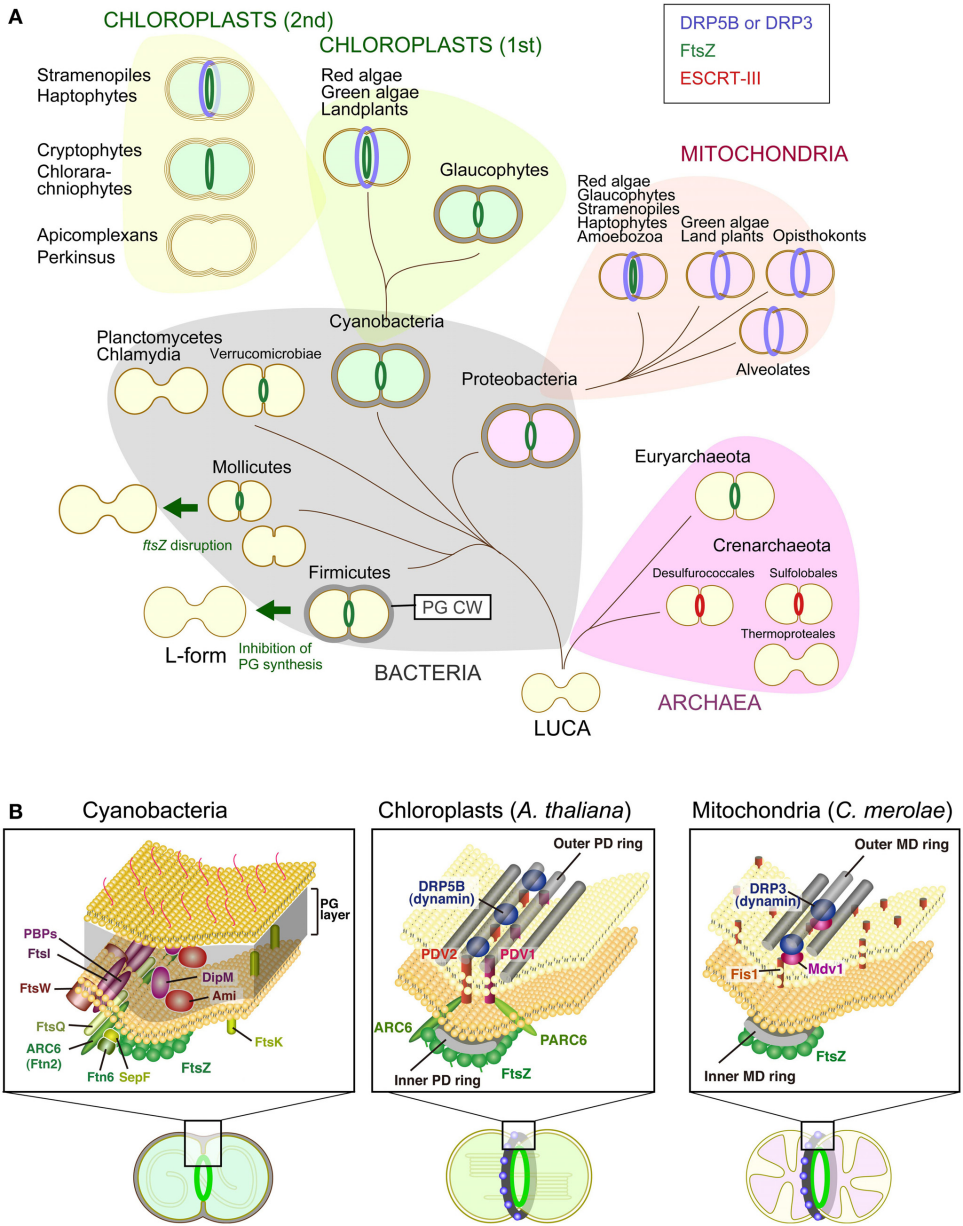
**Variation in the division machinery of prokaryotic cells, mitochondria, and chloroplasts including non-photosynthetic plastids throughout the three domains of life. (A)** Cells of the last universal common ancestor (LUCA) probably did not have extracellular envelopes, such as a PG cell wall (PG CW) and proteinous S-layer, and cell division was likely performed by mechanical mode. Complex cellular envelope and division machinery evolved later. FtsZ-based division machinery appeared either in the common ancestor of Bacteria and Archaea or the common ancestor of Bacteria (in this scenario, FtsZ was later horizontally transferred to the Euryarchaeota). ESCRT-III-based division machinery appeared in the common ancestor of the Crenarchaeota. Some of the bacterial lineages, especially parasitic bacteria, have lost the FtsZ-based division machinery, likely because of a loss of the PG cell wall by regressive evolution. A portion of the FtsZ-based division machinery was transmitted to mitochondria and chloroplasts through endosymbiotic events. Two different DRPs were later integrated into the mitochondrial (DRP3) and chloroplast (DRP5B) division machinery. Chloroplasts were further transmitted to a wide array of eukaryotes by secondary endosymbiotic events of an ancestral red alga and green alga. **(B)** Comparison of the cyanobacterial, chloroplast and mitochondrial division machinery. For the cyanobacterial division machinery, a tentative diagram is shown (for the details, see Marbouty et al., 2009). The localization of Ftn2, SepF, FtsZ, and Ftn6 at the division site was determined experimentally. The localization of FtsE, FtsI, FtsK, FtsQ, and FtsW has not been determined in cyanobacteria, but these proteins are involved in the division machinery of other bacterial species. For the chloroplast and mitochondrial division machinery, components in the land plant *A. thaliana* and red alga *C. merolae* are shown, respectively. Only the known, division site-localized components are shown. The localization of Fis1 has not been determined in *C. merolae*, but Fis1 is involved in the recruitment of the dynamin-related protein to the mitochondrial division site in *Saccharomyces cerevisiae* (Kiefel et al., 2006).

Mitochondria and chloroplasts (including non-photosynthetic plastids in land plants and parasitic protists) arose as a consequence of a series of endosymbiotic events more than one billion years ago. Mitochondria first arose from an alpha-proteobacterial ancestor that was integrated into a primitive eukaryotic host cell. Chloroplasts later arose from a cyanobacterial ancestor acquired by a eukaryote in which mitochondria were already established (Figure 1) (Reyes-Prieto et al., 2007; Keeling, 2013). Reminiscent of their free-living bacterial ancestors, these organelles possess their own genomes and machinery for expressing genomic information (e.g., nucleoids and ribosomes). In addition, mitochondria and chloroplasts multiply by the division of pre-existing organelles (Kiefel et al., 2006; Kuroiwa et al., 2006; Miyagishima et al., 2011; Yoshida et al., 2012; Osteryoung and Pyke, 2014). However, the chloroplast genome does not contain sufficient information for carrying out division, indicating that nuclear genome perform and regulate the chloroplast division.

Structural and molecular genetic studies have shown that chloroplast division is performed by constriction of a ring-like nucleus-encoded protein complex which encompasses both the inside and the outside of the inner and outer envelope membrane. Most of the components that function on the stromal side are descended from the cell division machinery of a cyanobacterial ancestor of chloroplasts, in which the self-assembling GTPase FtsZ plays a pivotal role. In contrast, all of the known components that function on the cytosolic side were added by the eukaryotic host cell subsequent to the endosymbiotic event, in which another self-assembling GTPase, dynamin-related protein (DRP5B) has a role (Miyagishima et al., 2011; Yoshida et al., 2012; Osteryoung and Pyke, 2014) (Figure 1). Bacterial FtsZ self-assembles into rings inside of liposomes and induces constrictions of these liposomes *in vitro* (Osawa et al., 2008). The helical self-assembly of dynamin tabulates liposomes, and disassembly of the helix results in membrane fission *in vitro* (Roux and Antonny, 2008). Thus, FtsZ and DRP5B probably participate in the generation of constrictive force in chloroplast division.

**Figure 2:**
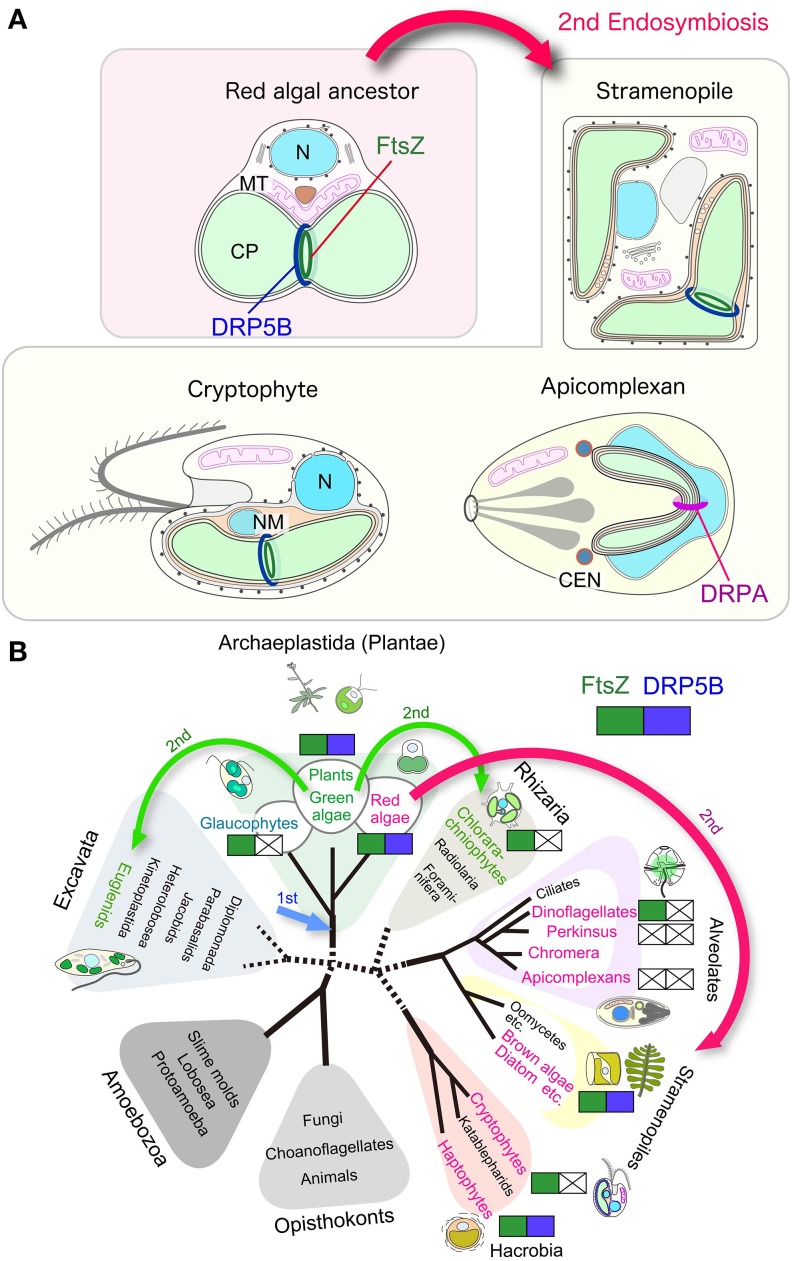
**Variation in the division machinery of chloroplasts and non-photosynthetic plastids throughout the eukaryotic phylogenetic tree. (A)** Schematic view of the chloroplast and non-photosynthetic plastid division machinery in red algae, stramenopiles, cryptophytes, and apicomplexans. Components other than FtsZ and DRPs are not shown. **(B)** Distribution of cyanobacteria-descended chloroplast division FtsZ and eukaryotic-host derived DRP5B. Red algae and groups containing chloroplasts or non-photosynthetic plastids of red algal origin are shown in red. Viridiplantae (green algae and land plants) and groups containing chloroplasts of green algal origin are shown in green. Arrows indicate the primary endosymbiotic event of a cyanobacterium (1st) and secondary endosymbiotic events (2nd). N, nucleus; MT, mitochondrion, CP, chloroplast, NM, nucleomorph.

A number of studies have reported that the inactivation of certain components of the division machinery impairs chloroplast and non-photosynthetic plastid division. However, several exceptions have been reported. (1) Non-photosynthetic plastids in land plants are able to proliferate in FtsZ depleted-cells (Schmitz et al., 2009) or FtsZ ring-deficient cells (Chen et al., 2009). (2) In DRP5B-depleted cells, non-photosynthetic plastids and chloroplasts are able to proliferate although the efficiency of chloroplast division is compromised (Robertson et al., 1996; Sakaguchi et al., 2011). (3) Chloroplasts in glaucophytes, which have a peptidoglycan (PG) layer between the inner and the outer envelope membrane in a manner like bacterial cells and, unlike chloroplasts of other eukaryotic lineages, divide without DRP5B (Miyagishima et al., 2014). (4) The genomes of some of the parasitic eukaryotes that carry non-photosynthetic plastids do not encode FtsZ or DRP5B, or both (Figure 2) (Van Dooren et al., 2009). Although it is not known what kind of molecular mechanisms are involved in such FtsZ- and/or DRP5B-less chloroplast or non-photosynthetic plastid division at this point, insight into how chloroplasts are able to divide in the absence of FtsZ and/or DRP would lead to a better understanding of the function and evolution of chloroplast division machinery and the as-yet-unknown mechanisms that facilitate chloroplast division in conjunction with the constriction of the division machinery.

This review aims to summarize the currently available information on chloroplast or non-photosynthetic plastid division in FtsZ or DRP5B mutants, as well as chloroplast- or non-photosynthetic plastid-carrying eukaryotes that do not possess FtsZ and/or DRP5B, to facilitate characterization and understanding the mechanisms of FtsZ- and/or DRP5B-less chloroplast division in the future. We will introduce information on prokaryotic cell division first because recent studies have shown that the existence of alternative mechanisms for cell division in *ftsZ* knockouts in certain bacterial lineages under specific conditions and in bacterial lineages that have lost the *ftsZ* gene by regressive evolution (Bernander and Ettema, 2010; Erickson and Osawa, 2010). Because localization of FtsZ is the first event that occurs at the division site in chloroplasts (Miyagishima et al., 2011; Yoshida et al., 2012; Osteryoung and Pyke, 2014) and bacteria (De Boer, 2010; Egan and Vollmer, 2013), and FtsZ is essential for the recruitment of other components of the division machinery, cell division in the *ftsZ* knockout does not utilize conventional division machinery. In bacteria in which the genome does not encode FtsZ and also lacks most of the other known components of division machinery, cell division should be performed by significantly different (but as-yet-unknown in most cases) mechanisms from the well-understood FtsZ-based division machinery. In addition, in a group of Archaea, it has been shown that a protein complex similar to the eukaryotic Endosomal Sorting Complexes Required for Transport (ESCRT)-III complex, which has a key role in membrane remodeling in eukaryotes, including cytokinesis, is involved in cell division instead of the FtsZ-based system (Lindas et al., 2008; Makarova et al., 2010; Koonin and Mulkidjanian, 2013) (Figure 1). Such information can provide certain important hints for understanding possible alternative mechanisms for chloroplast and non-photosynthetic plastid division. Then we briefly introduce the mitochondrial division machinery in addition to the chloroplast division machinery, because another dynamin-related protein, DRP3, is involved in mitochondrial division. Furthermore, in some eukaryotic lineages, FtsZ of alpha-proteobacterial origin is involved in the division process in addition to DRP3 (Kiefel et al., 2006; Kuroiwa et al., 2006) (Figure 1). Because the dispensability of FtsZ in bacterial cell division is often related to either the evolutionary or experimental loss of the PG layer (Figure 1), the information on the possible PG synthesis in chloroplasts is also summarized. After that, we will summarize what is known about FtsZ- and/or DRP5B-less chloroplast as well as non-photosynthetic plastid division.

### Bacterial cell division based on FtsZ and PG synthesis

Most bacterial cell possesses a cell wall outside the cytoplasmic membrane which protects them against mechanical stress and allows them to cope with high internal osmotic pressure. The bacterial cell wall outside the cytoplasmic membrane is constituted of a PG layer, proteinous S-layer and, in the case of Gram-negative bacteria, an outer membrane (Albers and Meyer, 2011; Egan and Vollmer, 2013). Among them, the PG layer, which consists of glycan strands cross-linked by covalently linked peptides, is important for withstanding the internal osmotic pressure of bacterial cells and maintains their shape. This explains why a wide array of antibiotics, such as the beta-lactams, target the PG biosynthetic pathway. Thus, bacterial cell division has to coordinate PG growth and remodeling with membrane remodeling (Collier, 2010). This requirement is evident from the fact that several cell division proteins, including FtsZ, are encoded together with the enzymes involved in the PG synthesis in the same genomic region, which is called the Division and Cell Wall (DCW) cluster in bacteria (De Boer, 2010; Egan and Vollmer, 2013).

Cell division in most bacteria is performed by the concerted activity of ~20 proteins that assemble into a division machinery complex at the division site (Figure 1B). The division site is established by polymerization of the tubulin-like FtsZ GTPase into a ring structure that acts as a scaffold for the assembly of other components. At the first stage, FtsZ associates with factors that stabilize FtsZ polymers, such as Ftn2 and SepF in cyanobacteria and FtsA, ZipA, and ZapA in *E. coli*, and tether them to the cytoplasmic membrane. After a marked delay (17 min/the *E. coli* cell cycle of 85 min) (Aarsman et al., 2005), a second set of proteins is recruited directly or indirectly by the FtsZ ring, which includes FtsQ, FtsW, PBP1, FtsI, DipM, and Ami proteins to form the mature constriction-competent division machinery. The precise function of FtsQ is not known. FtsW functions as lipid II (precursor of PG) flipase. PBP1 polymerizes lipid II to PG strand and FtsI (PBP3) crosslinks PG strand. DipM and Ami amidase are involved in PG degradation at the division site. After the maturation of the divisiome, the PG layer concurrently grows inward along with membrane invagination (De Boer, 2010; Egan and Vollmer, 2013). The PG is hydrolyzed to constrict the outer membrane in the case of Gram-negative bacteria and split daughter cells, which share the PG layer at the site of the septum (Collier, 2010). Although several models have been proposed but it is still unclear how PG synthesis is activated by the division machinery and how the PG synthesis and degradation is coordinated at the division site.

### FtsZ-independent cell division in the PG-deficient L-form of *B. subtilis*

By suppressing PG synthesis with either antibiotics or gene-manipulation and long-term passage on osmotically supportive medium to prevent cell lysis, many bacteria change to (completely or partially) cell wall-deficient “L-form” state (Allan et al., 2009). L-form bacteria are capable of growth and cell division. The transition to the L-form has been known to require one or more genetic changes (Allan et al., 2009). *E coli* (gamma-Proteobacteria; Gram-negative) L-forms still retain residual PG synthesis, which is essential for growth, and the FtsZ ring is probably involved in L-form cell division (Allan et al., 2009). In contrast, *B. subtilis* (Firmicutes; Low GC Gram-positive) L-forms proliferate without the formation of a normal FtsZ ring, even when PG synthesis is completely blocked. In addition, *B. subtilis* L-forms are able to proliferate even when FtsZ is depleted. These L-forms multiply in a manner accompanied by shape perturbations, including blebbing, tubulation and vesiculation (Leaver et al., 2009). This “biophysical” mode of division in a *B. subtilis* L-form turned out to have been acquired by a mutation which causes excess membrane synthesis and increases the cell surface to volume ratio. In addition, increased lipid synthesis by the overexpression of Acetyl-CoA carboxylase enabled wild-type protoplasts to multiply in a manner similar to the L-form (Mercier et al., 2013).

These observations raise the possibility that cell division in wall-deficient cells, including ancestral prokaryotic cells, does not require elaborate cell division machinery at the division site and instead occurs spontaneously by an increase of the cell surface to volume ratio resulting from an acceleration of cytoplasmic membrane growth or a reduction of cell volume by evaporation (Koonin and Mulkidjanian, 2013; Mercier et al., 2013).

### FtsZ-less cell division in mollicutes, which lack a PG cell wall

The Mollicutes are parasitic or saprotrophic bacteria with a very reduced genome that lack a PG cell wall, but have a cell membrane contains sterol. However, the cells often possess a certain characteristic shape and the ability to adhere and locomote over solid surfaces. Mollicutes evolved from bacteria of the PG-walled Firmicutes (Low-GC Gram-positive bacteria) by regressive evolution with reduction of their genomes, probably because of their parasitic life in niches with constant environments (Figure 1) (Wolf et al., 2004). The best-studied group in the Mollicutes is the Mycoplasma. The DCW cluster in most of the sequenced Mycoplasma includes only *mraZ, mraW*, and *ftsZ* (Alarcón et al., 2007), consistent with the lack of PG. Moreover, some species even lack the *ftsZ* gene (Bernander and Ettema, 2010). Disruption of the *ftsZ* gene in *Mycoplasma gentitalium* is not lethal and the mutant cell is able to divide by motile activity in which the two halves of the cell migrate in opposite directions (Lluch-Senar et al., 2010). As in the case of the Mycoplasma, the genome of other Mollicutes, such as Spiroplasma and Ureaplasma lack *ftsZ* (Table 1) (Bernander and Ettema, 2010).

**Table 1:** **Distribution of FtsZ and the mechanism of cell division in prokaryotes**.

**Phylum/Class**	**Species**	**FtsZ**	**Cdv**	**PG^a^**	**Division^b^**	**Notes**
**ARCHAEA, CRENARCHAEOTA**						
Thermoproteales	*Thermoproteus tenax*	–	–	–		Actin-like protein is likely involved in the cell division.
Desulfurococcales	*Aeropyrum pemix*	–	+	–	ESCRT	
Sulfolobales	*Sulfolobus acidocaldarius*	–	+	–	ESCRT	
**ARCHAEA, EURYARCHAEOTA**						
Methanosarcinales	*Methanosarcina mazei*	+	–	–	FtsZ	
Halobacteriales	*Haloquadratum walsbyi*	+	–	–	FtsZ	
Methanomicrobiales	*Methanoculleus marisnigri*	+	–	–	FtsZ	
Thermoplasmatales	*Thermoplasma volcanium*	+	+	–		
Methanococcales	*Methanopyrus kandleri*	+	–	–	FtsZ	
Thermococcales	*Pyrococcus furlosus*	+	–	–	FtsZ	
**ARCHAEA, THAUMARCHAEOTA**						
Thaumarchaeota	*Nitrosopumilus maritimus*	+	+	–	ESCRT	
**ARCHAEA, NANOARCHAEOTA**						
Nanoarchaeota	*Nanoarchaeum equitans*	+	–	–	FtsZ	
**ARCHAEA, KORARCHAEOTA**						
Korarchaeota	*Korarchaeum cryptofilum*	+	–	–	FtsZ	
**BACTERIA**						
Firmicutes	*Bacillus. subtilis*	+	–	+	FtsZ	The L-form divides without FtsZ.
Mollicutes	*Mycoplasma genitalium*	+	–	–	FtsZ	*ftsZ* knockouts divide by locomotion.
Mollicutes	*Mycoplasma mobile*	–	–	–		Cells are likely able to divide by locomotion.
Mollicutes	*Ureaplasma urealyticum*	–	–	–		Cells are likely able to divide by locomotion.
Mollicutes	*Phytoplasma* sp.	–	–	–		Cells are likely able to divide by locomotion.
Verrucomicrobiae	*Verrucomicrobium spinosum*	+	–	–	FtsZ	
Chlamydiae	*Chlamydia trachmotis*	–	–	+	MreB	The PG ring forms at the mid cell.
Planctomycetes	*Planctomyces limnophilus*	–	–	–		Cells multiply by budding.
**BACTERIAL ENDOSYMBIONTS**						
γ-proteobacteria	*Ruthia magnifica*	–	–			Endosymbiont in the gut of a giant clam.
γ-proteobacteria	*Vesicomyosocius okutanii*	–	–			Endosymbiont in a deep-sea clam.
γ-proteobacteria	*Carsonella ruddi*	–	–			Endosymbiont in a psyllid.
α-proteobacteria	*Hodgkinia cicadicola*	–	–			Endosymbiont in a cicada.
Bacteroidetes	*Sulcia muelleri*	–	–			Endosymbiont in a glassy-winged sharpshooter.

a +, PG has been detected; –, PG has not been detected; Blank, unknown.

b Blank, unknown.

It is plausible that the PG layer was lost in Mollicutes because of their parasitic form of life under a constant osmotic pressure. The ancestor of Mollicutes had established a cell division mechanism based on both FtsZ and motile activity on a solid surface. During the course of regressive evolution, FtsZ was independently lost in certain species, because they are able to divide by locomotion-based mechanisms. This situation is similar to “traction-mediated cytokinesis” or “cytokinesis B” in the eukaryotic slime mold *Dictyostelium discoideum*, in which cells can divide by the migration of daughter cells in opposite directions on a solid surface even when the actomyosin-based contractile ring is inactivated (Uyeda and Nagasaki, 2004).

### Cell division in the chlamydiae with no FtsZ encoded

The Chlamydiae is a bacterial phylum in which the members are obligate intracellular parasites. Some chlamydial species encode a complete set of genes for PG synthesis and are thus sensitive to PG-targeting beta-lactam antibiotics. Nevertheless, attempts to detect or purify PG in Chlamydiae have been unsuccessful and this situation has been called the “chlamydial anomaly” (Mohammadi and Breukink, 2014). However, recent studies using electron cryotomography, mass spectrometry as well as *in situ* fluorescent labeling demonstrated that some species synthesize a unique type of PG which localizes to the division site (Pilhofer et al., 2013; Liechti et al., 2014). Further, it was shown that PBP2 and PBP3 (FtsI), which are involved in PG synthesis in other bacteria, are required for cell division in an MreB (bacterial actin homolog)-dependent manner in *Chlamydia trachmotis* (Ouellette et al., 2012). In the Chlamydiae *Waddlia chondrophila*, RodZ (a regulator of MreB) and MreB localize at the division site, and biosynthesis of PG precursor lipid II is required for the recruitment of RodZ to the division site (Jacquier et al., 2014).

Thus, the structure and distribution of PG have changed over the course of evolution, and the FtsZ-based division mechanism was probably replaced by the MreB-dependent system in an ancestral form of the Chlamydiae.

### FtsZ-less cell division in the planctomycetes-verrucomicrobiae-chlamydiae superphylum

Phylogenetic studies have shown that the bacterial phyla Planctomycetes, Chlamydiae (described above), Lentisphaerae and Verrucomicrobiae are monophyletic (i.e., a PVC superphylum) and have branched in this order (Figure 1) (Gupta et al., 2012). Planctomycetes are aquatic free-living bacteria which reproduce by budding (Fuerst, 1995). Planctomycetes lack PG and their walls are instead composed of glutamate-rich glycoprotein (Fuerst, 1995). As in the case of the Chlamydiae, *ftsZ* (as well as many other genes) is absent from the DCW cluster in the genomes of Planctomycetes (Pilhofer et al., 2008). In contrast, all of the sequenced Verrucomicrobiae genomes to date contain *ftsZ* (Pilhofer et al., 2008), whereas the Verrucomicrobiae also lack the typical PG cell wall (Yoon, 2011). A previous phylogenetic analysis suggested that *ftsZ* was lost independently in Chlamydiae and Planctomycetes. However, it is currently unknown how cell proliferation by budding is carried out in Planctomycetes (Pilhofer et al., 2008).

### FtsZ-less cell division in bacterial endosymbionts in eukaryotes

In a manner similar to certain parasitic bacteria, several obligate endosymbiotic bacteria have undergone genome reduction (McCutcheon and Moran, 2012) and lost the *ftsZ* gene (Table 1) (Bernander and Ettema, 2010). For example, *Carsonella ruddii* is an obligate endosymbiotic gamma proteobacterium in psyllid cells, which possesses the smallest known genome in bacteria (112 kb) and lacks *ftsZ* and other known components of the bacterial division machinery. But the tubular cells somehow proliferate in accord with the proliferation of bacteriocyte cells which accommodate *C. ruddii* (Nakabachi et al., 2006). There likely is a linkage between the loss of the PG cell wall and loss of *ftsZ* in these *ftsZ*-less bacterial endosymbionts, because they apparently do not require a rigid cell wall in osmotically stable host cells. However, there has been little information on the structure and/or composition of the cell surface in these endosymbiotic bacteria.

### Archaeal cell division: FtsZ, ESCRT-III, or absence of the both

Currently, the Archaea are subdivided into the Euryarchaeota, Crenarchaeota, Thaumarchaeota, Nanoarchaeota, and Korarchaeota. Of the five phyla, only a few species have been identified in the last three types (Albers and Meyer, 2011). The cell surface in most archaeal species possesses a proteinous S-layer, as in bacteria, but these species do not possess PG, except that PG-like polymers have been detected in some of the Euryarchaeota (Albers and Meyer, 2011). However, the cell envelope contains other types of polysaccharides, methanochondroitin, or proteinous sheaths in addition to the S-layer, depending on the lineage (Albers and Meyer, 2011). The genomes of Thaumarchaeota, Nanoarchaeota, Korarchaeota and almost all of the members of the Euryarchaeota encode FtsZ, and are believed to possess bacterial-type division machinery (Table 1). In contrast, *ftsZ* is missing from all (17 species) of the fully sequenced genomes of the Crenarchaeota (Table 1) (Makarova et al., 2010).

A recent study showed that, in *Sulfolobus acidocaldarius* (Crenarchaeota, Sulfolobales), the CdvA, CdvB, and CdvC proteins localize at the division site. CdvB and CdvC are related to components of the eukaryotic ESCRT-III protein complex, although CdvA appears to be uniquely present in the Crenarchaeota (Lindas et al., 2008). In eukaryotes, the ESCRT complex function in membrane fission process, such as multivesicular body formation, cytokinesis, and separation of envelope viruses from the plasma membrane (Schiel et al., 2013). Cdv proteins are conserved in Sulfolobales and Desulfococales (both belong to Crenarchaeota) and are encoded in the genomes of certain Euryarchaeotes (Table 1) (Makarova et al., 2010). These results suggest that cell division in Sulfolobales and Desulfococales is performed by a system related to the eukaryotic ESCRT-III machinery. The genome of the Thaumarchaeon *Nitrosopumilus maritimus* encodes both the FtsZ and Cdv proteins (Makarova et al., 2010), but a recent study has suggested that the cells likely divide using Cdv and not FtsZ, based on the localization of the Cdv proteins, but not FtsZ, to the division sites (Busiek and Margolin, 2011; Pelve et al., 2011).

The genomes of the Thermoproteales (Crenarchaeota) lack both the *ftsZ* and *cdv* genes (Makarova et al., 2010). In contrast to the gradual invagination of the cytoplasmic membrane and the surrounding cell wall materials in most cases of prokaryotic cell division, it has been observed that Thermoproteales proliferate by a rapid snapping off of elongated cells (Horn et al., 1999). The nature of the division machinery is currently unknown, but actin-like proteins are likely involved in the process because the Thermoproteales possess a conserved operon containing a gene encoding a protein which is most closely related to eukaryotic actin and actin-like proteins (Makarova et al., 2010).

### Chloroplast division machinery

Chloroplast division is accomplished by the constriction of ring structures at the division site, which encompasses both the inside and the outside of the two envelopes. A part of the division machinery inside the chloroplast (i.e., the inner envelope and its stromal side) is descended from the cyanobacterial division machinery based on FtsZ. In contrast, other parts of the division machinery in the outer envelope and its cytosolic side involve proteins specific to eukaryotes, including a member of the dynamin family of GTPases, DRP5B (Miyagishima et al., 2011; Yoshida et al., 2012; Osteryoung and Pyke, 2014). In addition, the division machinery involves a bundle of glucan filaments, called the outer PD ring, on the cytosolic side of the outer envelope membrane (Yoshida et al., 2010) (Figure 1B). Proteins distantly related to the eukaryotic dynamin family have been found in several species of bacteria, thus the common ancestor of the eukaryotic dynamin family is likely of bacterial origin. The bacterial dynamin-like proteins have been shown to form an oligomer (Low and Lowe, 2006) and mediate membrane fusion *in vitro* (Burmann et al., 2011), while the function of these proteins *in vivo* in terms of membrane remodeling is not yet known.

While the cyanobacterial genomes encode a single FtsZ protein, two phylogenetically and functionally distinct FtsZ proteins have evolved in algae and plants by gene duplication and differentiation (Osteryoung et al., 1998; Miyagishima et al., 2004; Terbush and Osteryoung, 2012; Osteryoung and Pyke, 2014). Both proteins colocalize on the stromal side of the division site (McAndrew et al., 2001; Kuroiwa et al., 2002). FtsZ2 in Viridiplantae (green algae and land plants) has retained a short C-terminal domain (Osteryoung and Mcandrew, 2001; Miyagishima et al., 2004) that is essential for binding to the inner envelope-spanning protein ARC6 (Maple et al., 2005), which positively regulates FtsZ polymerization at the division site (Vitha et al., 2003). In contrast, FtsZ1 lacks the C-terminal motif (Osteryoung and Mcandrew, 2001; Miyagishima et al., 2004) and does not interact with ARC6 (Maple et al., 2005).

Studies in the seed plant *Arabidopsis thaliana* and the unicellular red alga *Cyanidioschyzon merolae* suggest that the plastid division complex is assembled in a direction from the inside to the outside of the chloroplast division site before the onset of constriction, in the order of the FtsZ ring, the inner PD ring of unknown composition, the outer PD ring and the DRP5B ring. The stromal division complex assembles independently of the cytosolic complex, whereas the assembly of the cytosolic complex depends on the stromal complex.

The studies on protein-protein interactions and localization of the division complex components in plastid division mutants in *A. thaliana* have suggested the following scheme. (1) FtsZ ring formation is promoted by the inner-envelope-spanning protein ARC6, which is descended from a cyanobacterial ancestor of chloroplasts (Vitha et al., 2003). (2) The outer envelope spanning proteins PDV1 and PDV2, which are specific to land plants, are recruited to the division complex by ARC6 and PARC6 (a paralog of ARC6) (Glynn et al., 2008, 2009). (3) PDV1 and PDV2 recruit cytosolic DRP5B (Miyagishima et al., 2006; Holtsmark et al., 2013) and then the division complex starts to constrict. Although the relationship between these proteins and the PD ring has not been characterized in *A. thaliana*, formation of the PD ring is preceded by that of the FtsZ ring before the onset of constriction in other land plants (Kuroiwa et al., 2002). In the red alga *C. merolae*, the FtsZ, inner PD, outer PD and DRP5B rings form in this order (Miyagishima et al., 2003). In addition, DRP5B is recruited to the division site in an outer PD ring-dependent manner (Yoshida et al., 2010). This is consistent with the presence of the PD ring at the constricted region of giant chloroplasts in the *A. thaliana arc5* (*drp5B*) mutant (Robertson et al., 1996).

### Mitochondrial division machinery

As in the case of chloroplast division, mitochondrial division in certain eukaryotic lineages involves the nucleus-encoded FtsZ descended from alpha-proteobacterial mitochondrial ancestor, and DRP-based division machinery (Figure 1). In addition, the MD ring, which is a structure similar to the outer PD ring, has been observed in the red alga *C. merolae*, the true slime mold *Physarum polycepharum* and the stramenopile *Nannochloropsis oculata* by transmission electron microscopy (Kuroiwa et al., 2006). FtsZ localizes on the matrix side of the inner membrane, whereas DRP localizes to the cytosolic side of the outer membrane (Kiefel et al., 2006; Kuroiwa et al., 2006; Miyagishima et al., 2011). As in the case of chloroplast division, the FtsZ, MD and dynamin rings form at the division site in this order in the red alga *C. merolae* (Nishida et al., 2003; Miyagishima et al., 2011). However, whereas mitochondrial DRP appears to have been conserved in eukaryotes, the FtsZ involved in mitochondrial division has been lost independently several times during the course of eukaryotic evolution (Figure 1). Nucleus-encoded mitochondrial FtsZ has been found in genomes of amoebozoans, glaucophtes, red algae, stramenopiles (diatoms and brown algae) and haptophytes, whereas it is absent from eukaryotes such as opistokonts (fungi and animals), green algae and land plants (Kiefel et al., 2006).

The basis for the presence or absence of mitochondrial FtsZ is currently unclear. However, one possibility is that the generally more bacterium-like mitochondrial morphology of FtsZ-containing organisms is well-suited to FtsZ-based division, whereas reticulated mitochondria that constantly fuse and divide may dispense with an FtsZ-based mechanism of division (Kiefel et al., 2006).

### The relationship between cyanobacterial PG synthetic enzyme homologs and chloroplast division

As described above, one of the primary functions of the FtsZ-based division machinery in bacteria is to promote the synthesis of the PG layer at the division site during cell division. Consistent with this primary function, in some but not all cases, the loss of the PG cell wall by either regressive evolution or by experimental manipulation correlates with the loss or dispensability of the FtsZ-based division machinery. In contrast to this situation, chloroplast division (and mitochondrial division in some eukaryotic lineages) is performed by FtsZ- and DRP5B-based division machinery although it is generally believed that chloroplasts and non-photosynthetic plastids (and mitochondria) lack a PG layer except in glaucophytes, because PG has not been detected by any of the conventional methods. One possible explanation for this is that the primary function of FtsZ has been changed to recruiting cytosolic chloroplast division proteins of eukaryotic host origin, rather than proteins for PG synthesis and degradation, during the course of the loss of the PG layer.

However, the genes for PG synthesis are encoded in the genomes of various lineages of algae and land plants besides glaucophytes (Takano and Takechi, 2010). For example, the nuclear genomes of the moss *Physcomitrella patens* and the fern (lycophyte) *Selaginella moellendorffii* encode homologs of all of the proteins necessary for the PG biosynthetic pathway in bacteria (MurA to G, Ddl, MraY, and PBP proteins) (Takano and Takechi, 2010). The chloroplast genome of some green algae (Prasinophyceae) and charophyte algae encodes FtsI and FtsW (Miyagishima et al., 2012). In the charophytes, the *Closterium peracerosum–strigosum–littorale* complex, the moss *P. patens*, and the fern *Selaginella nipponica*, it has been reported that PG-targeting antibiotics (inhibitors of MurA, Ddl, and PBP) impair chloroplast division (Kasten and Reski, 1997; Katayama et al., 2003; Matsumoto et al., 2012). In addition, the nucleus-encoded MurE, PBP and MraY were confirmed to be targeted to *P. patens* chloroplasts (Machida et al., 2006; Homi et al., 2009) and depletion of MurE, MurA and MurY inhibited chloroplast division in *P. patens* (Machida et al., 2006; Homi et al., 2009).

On the other hand, the genome of the green alga (Chlorophyceae) *Chlamydomonas reinhardtii* encodes only a homolog of MurE, while the *A. thaliana* genome encodes homologs of some of the PG-synthetic proteins, but does not encode MurA, MurB, MurC, or MurD (Takano and Takechi, 2010). In addition, the gene disruption analysis suggests that MurE is required for chloroplast development but not for chloroplast division in *A. thaliana* (Garcia et al., 2008).

In bacterial cell division, PG hydrolysis at the division site is also required (De Boer, 2010; Egan and Vollmer, 2013). Similar to genes encoding proteins of the PG synthetic pathway, homologs of DipM, which hydrolyze PG at the division site in bacteria, are encoded in the genomes of several lineages of algae and land plants (Miyagishima et al., 2014). In addition, disruption of *DipM* genes inhibited chloroplast division in *P. patens* (Miyagishima et al., 2014).

These observations suggest the possibility that PG was lost more recently than previously believed (from the common ancestor of red and green algae and land plants after the glaucophytes branched) and that the loss of PG from chloroplasts has occurred multiple times independently in green algae, red algae, and land plants (Takano and Takechi, 2010). This may be the reason for the retention of FtsZ-based division machinery in chloroplasts, and the division machinery likely plays a role to coordinate PG synthesis and chloroplast division in some lineages of algae and plants.

### The possibility of non-photosynthetic plastid division in *ftsZ*-knockouts of land plants

Stable knockout lines of a single *ftsZ* gene have been generated in the moss *Physcomitrella patens* (Strepp et al., 1998; Martin et al., 2009) and the seed plant *A. thaliana* (Yoder et al., 2007; McAndrew et al., 2008; Schmitz et al., 2009). In both cases, mutant cells contain larger and fewer plastids than the wild type as a result of an impairment of chloroplast division. However, each leaf cell still contains at least a few enlarged plastids, suggesting that chloroplast and/or non-photosynthetic plastid division are not completely blocked in these knockout lines. One possible reason for that is the existence of multiple *ftsZ* genes in plant genomes resulting in functional redundancy among them because the *A. thaliana* genome encodes a single FtsZ1 and two FtsZ2 (FtsZ2-1 and FtsZ2-2) proteins and the *P. patens* genome encodes five FtsZ proteins. A molecular genetic study showed that *FtsZ2-1* and *FtsZ2-2* in *A. thaliana* are functionally redundant (Schmitz et al., 2009). However, a double knockout line of *FtsZ2-1* and *FtsZ2-2* and a triple knockout line of all of the *FtsZ* genes (*FtsZ1*, *FtsZ2-1*, and *FtsZ2-2*) of *A. thaliana* displayed no drastic defects in growth or fertilization and the mesophyll cells still contained one or two enlarged chloroplasts (Schmitz et al., 2009).

In algae and mosses, chloroplasts are usually the only type of plastid present. In contrast, vascular plants have evolved a plastid differentiation system in which all of the plastids, including chloroplasts, chromoplasts, leucoplasts, and amyloplasts, are derived from non-photosynthetic proplastids in meristematic cells (Lopez-Juez and Pyke, 2005). All of these types of plastids are capable of division (Possingham and Lawrence, 1983) and FtsZ rings are evident at least at the proplastid and chloroplast division sites (McAndrew et al., 2008; Okazaki et al., 2009). The relationship between chloroplast division and cell division in seed plants is also complex. For example, in spinach (Spinacia oleracea), the shoot apical meristem contains approximately 12 proplastids, the division of which keeps pace with cell division so that newly formed cells have essentially the same number of proplastids. During leaf development, cells expand without cell division (but with endoreduplication), yet the chloroplasts still continue to divide and the number of chloroplasts per cell eventually reaches approximately 200 (Possingham and Lawrence, 1983). Therefore, in the *A. thaliana FtsZ* triple knockout line, even when chloroplast division is blocked in young leaf cell, the cells are probably able to expand while maintaining the number of chloroplasts. However, in the mutant, at least the proplastids in the meristematic tissues somehow proliferate without FtsZ.

A mutation in *arc6* impairs FtsZ assembly (Vitha et al., 2003) and results in a severe defect in both chloroplast and proplastid division, while the mutant plant displays no drastic defects in growth or fertilization (Robertson et al., 1995; Pyke, 1999; Chen et al., 2009). The photosynthetic cells in *arc6* contain irregularly shaped non-green plastids along with enlarged chloroplasts. These thylakoid-less non-green plastids are apparently generated by protrusion and fragmentation of enlarged chloroplasts (Chen et al., 2009), suggesting that there is likely to be an alternative mechanism by which at least non-photosynthetic plastids are able to proliferate without FtsZ. In a manner similar to the *A. thaliana arc6* mutant, in the tomato chloroplast division mutant *suffulta*, enlarged chloroplasts degenerate and give rise to a wild type-like population of chromoplasts (non-green plastids) in ripe fruit by a process of plastid budding and fragmentation. In addition, some stomatal guard cells in the mutant contain non-green pleomorphic plastids in addition to green chloroplasts (Forth and Pyke, 2006).

The above described observations imply that non-photosynthetic plastids without thylakoids are able to divide without the need of any FtsZ-based division machinery. In addition, non-photosynthetic plastids often display pleiomorphic tubular morphology unlike chloroplasts (Osteryoung and Pyke, 2014). The non-photosynthetic protrusions from chloroplasts in *arc6* (Chen et al., 2009) and *suffulta* (Forth and Pyke, 2006) are probably the result of an increase in the surface to volume ratio of the mutant chloroplasts. Thus, at least the non-photosynthetic plastids likely divide by tabulation (an increase in the surface to volume ratio) and subsequent budding, as in the case of cell division without FtsZ in the *B. subtilis* L-form (Mercier et al., 2013). Further analyses of non-green plastids in the above mentioned mutants by time-lapse imaging will help elucidate how they are able to divide without using the conventional division machinery.

### Chloroplast division in glaucophytes, the chloroplasts of which have a PG layer between the two envelope membranes

In contrast to plastids in other eukaryotic lineages, chloroplasts of the glaucophyte algae possess PG layer between the inner and outer envelope membranes. Evolutionary studies suggest that the glaucophyte algae were the earliest to branch off from the common ancestor of Archaeplastida (Plantae *sensu stricto*), prior to the divergence of the Red algae and Viridiplantae (Reyes-Prieto et al., 2007; Keeling, 2013) (Figure 1A). Chloroplast division in the glaucophyte alga *Cyanophora paradoxa* involves FtsZ ring formation on the stromal side of the division site (Sato et al., 2007). However, the genome (Price et al., 2012) does not encode DRP5B (Miyagishima et al., 2014).

In glaucophyte chloroplast division, the inner envelope membrane starts to constrict earlier than the outer envelope membrane does, and this is accompanied by an ingrowth of the PG layer at the division site, reminiscent of the cell division of cyanobacteria. Therefore, the gap between the two envelopes at the division site becomes much larger than in other parts of the chloroplast in the glaucophytes (Iino and Hashimoto, 2003; Sato et al., 2009). To allow the outer envelope membrane to constrict, the PG layer at the division site has to be cut from the outermost site, as in bacterial cell division. A recent study suggested that a DipM protein of cyanobacterial origin is involved in this PG splitting in *C. paradoxa* (Miyagishima et al., 2014). Thus, it appears that DRP5B was integrated into the chloroplast division machinery in parallel with the loss of the PG layer and development of the division machinery, in which the inner and the outer envelope membranes constrict synchronously.

### Chloroplast and non-photosynthetic plastids in DRP5B *knockouts* of land plants

Thus far, knockout lines of DRP5B have been generated in the moss *P. patens* (Sakaguchi et al., 2011) and the seed plant *A. thaliana* (Miyagishima et al., 2006). The *P. patens* genome contains three *DRP5B* genes and the triple knockout of all of these *DRP5B* genes impaired chloroplast division. However, the mutant protonemal cells still contained 4–5 enlarged chloroplasts (~50 chloroplasts per wild-type cell) (Sakaguchi et al., 2011), suggesting that the chloroplasts somehow underwent division without DRP5B.

*A. thaliana* genome contains a single *DRP5B* (called *ARC5*) gene and the mutation (Robertson et al., 1996; Gao et al., 2003) or knockout (Miyagishima et al., 2006) of *ARC5* impairs chloroplast division. However, even in the absence of the ARC5 protein, leaf mesophyll cells still contain 5–10 enlarged chloroplasts with constrictions at the division site. Moreover, there is no detectable defect in proplastid division in the shoot apical meristem of the *arc5* mutant (Robertson et al., 1996; Pyke, 1999). Consistent with there being no defects in proplastid division in the *arc5* mutant, the ARC5 protein is not detected in the wild-type shoot apical meristem (Okazaki et al., 2009). Thus, at least the proplastids in seed plants are apparently divide without the aid of DRP5B protein. At present, it is still possible that another DRP is involved in proplastid division and also plays a role in chloroplast division in a manner partly redundant to ARC5. However, this is not likely, because DRP5A, which is most closely related to DRP5B in plants and algae, is not involved in plastid division (Gao, 2005) but rather, in cytokinesis (Miyagishima et al., 2008). Furthermore, defects in chloroplast or proplastid division have not been observed in mutants of the other DRPs in *A. thaliana*.

### Secondary chloroplast division with or without DRP5B

A diverse array of eukaryotic lineages possess chloroplasts or, in the case of parasites, non-photosynthetic plastids that were acquired through secondary endosymbiotic events in which a eukaryotic alga was integrated into another, previously non-photosynthetic eukaryotic cell (Figure 2B). The secondary endosymbiotic event of a red algal ancestor gave rise to chloroplasts or non-photosynthetic plastids in stramenopiles (diatoms, brown algae, etc.), haptophytes, cryptophytes, most of the photosynthetic dinoflagellates, and apicomplexan parasites. Euglenids and chrorarachniophytes possess chloroplasts of a green algal secondary endosymbiotic origin (Figure 2B). The question of exactly how many endosymbiotic events have given rise to this evident diversity remains unanswered (Reyes-Prieto et al., 2007; Keeling, 2013).

A few studies on stramenoplile chloroplast division and recent genome investigations suggest that a part of the secondary chloroplast division machinery in stramenopiles is descended from a red algal endosymbiont. Secondary chloroplasts are surrounded by three or four membranes (Figure 2A). The inner two membranes are descended from the inner and the outer envelopes of the primary chloroplast. The two additional membranes are thought to correspond to the plasma membrane of the engulfed alga and the phagosomal membrane of the host cell, respectively. The outermost membrane is connected with the outer nuclear envelope, either directly or indirectly, through a roughly formed ER (Reyes-Prieto et al., 2007; Keeling, 2013).

Putative chloroplast division FtsZ is encoded in the nuclear genomes of stramenopiles (Kiefel et al., 2006), haptophytes (Nishikawa et al., 2010), and dinoflagellates (EST clone from *Lingulodinium polyedrum*, GI: 556888185) (Figure 2B; Table 2). In the case of the cryptophyte *Guillardia theta*, FtsZ is not encoded in the nuclear genome, but in the nucleomorph (a remnant of the engulfed red algal nucleus) genome (Fraunholz et al., 1998; Curtis et al., 2012) (Figure 2B; Table 2). In the stramenopile *Thalassiosira pseudonana*, DRP5B of a red algal origin localizes at the chloroplast division site (Miyagishima, 2011). In several stramenoplile species, the outer PD ring has been observed on the outer side of the second innermost membrane, which corresponds to the position of the outer PD ring in primary chloroplasts (Hashimoto, 2005; Weatherill et al., 2007). Thus, the division of the inner pair of membranes in secondary chloroplasts involves at least a portion of the primary chloroplast division machinery that is descended from the engulfed red alga (Figure 2A). However, DRP5B is not encoded in the genomes of the cryptophyte *Guillardia theta* or chlorarachniophyte *Bigelowiella natans* (Figure 2B; Table 2) (Curtis et al., 2012). At present, there is no information on the cytology or molecular cell biology of the chloroplast division that takes place in cryptophytes and chlorarachniophytes.

**Table 2:** **Distribution of chloroplast division FtsZ and DRP5B in eukaryotes**.

**Phylum**	**Species**	**FtsZ**	**DRP5B**
Glaucophyta	*Cyanophora paradoxa*	+	–
Rhodophyta (red algae)	*Cyanidioschyzon merolae*	+	+
Chlorophyta (green algae)	*Chlamydomonas reinhardtii*	+	+
Embryophyta (land plants)	*Arabidopsis thaliana*	+	+
Stramenopila	*Thalassiosira pseudonana*	+	+
Haptophyta	*Emiliania huxleyi*	+	+
Cryptophyta	*Guillardia theta*	+	–
Chlorarachniophyta	*Bigelowiella natans*	+	–
Dinoflagellata	*Lingulodinium polyedrum*	+	N/D^a^
Perkinsozoa	*Perkinsus marinus*	–	–
Apicomplexa	*Toxoplasma gondii*	–	–

a FtsZ but not DRP5B was identified in the EST database of Lingulodinium polyedrum. The whole genome data is not available at present.

### Non-photosynthetic plastid division takes place in parasitic protists without FtsZ or DRP5B

Nucleus encoded, plastid-targeted FtsZ proteins have been identified in all chloroplast-carrying eukaryotes except for Apicomplexa and Perkinsozoa (Figure 2B, Table 2). Ciliophora (cilliates), Apicomplexa, and Perkinsozoa and Dinoflagellata are grouped into superphylum Alveolata (the order of branching is indicated in Figure 2B) (Reyes-Prieto et al., 2007; Keeling, 2013). Among the alveolates, ciliates do not possess chloroplasts or non-photosynthetic plastids, whereas some dinoflagellates possess photosynthetic chloroplasts of secondary or tertiary endosymbiotic origin (Figure 2B) (Reyes-Prieto et al., 2007; Keeling, 2013). In the Alveolatae, in addition to dinoflagellate chloroplasts, non-photosynthetic plastids have been identified in Apicomplexa (McFadden et al., 1996; McFadden, 2011) and Perkinsozoa (Stelter et al., 2007; Matsuzaki et al., 2008; Fernandez Robledo et al., 2011).

Apicomplexa are a large group of parasitic unicellular eukaryotes which includes malarial parasites. Apicomplexans possess a single non-photosynthetic plastid (called an apicoplast) with its own genome. The apicoplast originated from a red algal secondary endosymbiotic event and is surrounded by four membranes. Apicoplasts are believed to function in lipid and heme biosynthesis and are necessary for apicomplexan survival (McFadden et al., 1996; McFadden, 2011). The apicomplexan genomes do not encode FtsZ or DRP5B (Figure 2B; Table 2). Thus, the plastid division machinery of the red algal ancestor of the apicoplast was lost after the secondary endosymbiotic event. In *Toxoplasma gondii*, apicoplast division is tightly associated with nuclear and cell division, and is characterized by an elongated, dumbbell-shaped intermediate. The edges of the dividing apicoplast are closely linked to the centrosome, and the spindle is involved in the segregation of dividing/divided apicoplasts to daughter cells (Striepen et al., 2000). Although DRP5B is not encoded in the genome, another DRP, DrpA, which is unique to apicomplexans, localizes at the apicoplast division site and is required for the fission that occurs there (Van Dooren et al., 2009). DrpA probably localizes on the cytosolic side of the outermost of the four membranes, whereas the DRP5B of a red algal origin in stramenopiles probably localizes on the cytosolic side of the second innermost membrane, which is topologically equivalent to the cytosolic side of the outer envelope of the red algal ancestor that was integrated into stramenopiles (Figure 2A).

Perkinsozoans are parasitic unicellular eukaryotes that infect molluscs, at times leading to disease and mass mortality. *P. marinus* is the most notorious, causing perkinsosis in both wild and farmed oysters. Electronmicroscopy showed that a *P. olseni* cell possesses tiny organelles surrounded by four membranes (Teles-Grilo et al., 2007), as in the case of the apicoplast. In addition, plant-type ferredoxin (Stelter et al., 2007) and enzymes in the MEP pathway (Matsuzaki et al., 2008), which synthesize isoprenoids in chloroplasts and non-photosynthetic plastids in other eukaryotes, are encoded in the nuclear genome of *P. marinus.* The deduced amino acid sequences of these proteins contain an N-terminal bipartite targeting peptide, composed of a signal peptide to guide the polypeptide into the ER lumen and a subsequent transit peptide to deliver the mature protein into the plastid lumen (Stelter et al., 2007; Matsuzaki et al., 2008; Fernandez Robledo et al., 2011). These observations suggest that perkinsozoans possess non-photosynthetic plastids as in the case of the apicomplexans, although the non-photosynthetic plastid apparently has lost its DNA (Matsuzaki et al., 2008). Neither FtsZ nor DRP5B is encoded in the *P. marinus* genome (TIGR draft genome database, http://blast.jcvi.org/er-blast/index.cgi?project=pmg) (Figure 2B; Table 2), indicating that the plastids proliferate by an as-yet-unknown mechanism in perkinsozoans.

## Conclusion and perspectives

The chloroplast division machinery is a chimeric protein complex based on stromal FtsZ of cyanobacterial origin and cytosolic DRP5B of eukaryotic host origin. Many studies have shown that the machinery is required for proper chloroplast and non-photosynthetic plastid division. However, there are exceptions, in which chloroplast and/or non-photosynthetic plastids are able to divide in FtsZ or DRP5B knockout cells, or in which the genomes of chloroplasts or non-photosynthetic plastid-bearing eukaryotes do not encode FtsZ and/or DRP5B. To facilitate future studies on how chloroplasts or non-photosynthetic plastids are able to divide, we have reviewed the available information on FtsZ-less prokaryotic cell division and FtsZ- or DRP5B-less organelle division.

In many but not all cases in prokaryotes, the loss of the PG cell wall by either regressive evolution or experimental manipulation correlates with the loss of the FtsZ-based division machinery at the division site. In a few cases, alternative mechanisms for cell division, such as a tearing away of daughter cells by locomotion and budding by an increase of the cell surface to volume ratio, have been shown to promote bacterial cell division without FtsZ. This correlation seems consistent with the fact that one of the primary functions of the FtsZ-based division machinery is to promote the synthesis of the PG layer at the division site during cell division. However, chloroplasts, except for those in glaucophytes, are believed to have lost the PG layer during evolution, yet still utilize FtsZ-based division machinery on the stromal side. One possible explanation is that the primary function of FtsZ has changed to recruit cytosolic chloroplast division proteins of eukaryotic host origin, rather than proteins for PG synthesis and degradation, at the division site. However, the genes for both PG synthesis (Takano and Takechi, 2010) and degradation (Miyagishima et al., 2014) are encoded in the genomes of several lineages of algae and land plants. In these lineages, PG-targeting antibiotics (Kasten and Reski, 1997; Katayama et al., 2003; Matsumoto et al., 2012) or disruption of the PG synthesis genes (Machida et al., 2006) inhibits chloroplast division. This situation is similar to the “chlamydial anomaly,” which was recently resolved by the detection of PG by means of newly established, more ensitive methods. Thus, an examination of non-proteinous materials in the inter membrane space and the outer envelope would be expected to lead to an advance in the understanding of the function of the FtsZ-based division machinery in organelle division. In this regard, a recent study revealed that the outer PD ring in the red alga *C. merolae* is composed not of proteins, but of glucan strands.

In land plants, non-photosynthetic plastids apparently are able to divide in *FtsZ* knockout cells, even thought the FtsZ-based division machinery localizes at the plastid division site in the wild-type FtsZ ring formation is required for recruitment of the other components of the division machinery including DRP5B. Thus, in FtsZ knockout cells, non-photosynthetic plastids probably divide without the canonical division machinery. Non-photosynthetic plastids in land plants display tubular structures of a much smaller diameter than chloroplasts. Thus, non-photosynthetic proplastids in land plants may be able to divide by tubulation and budding, as in the case of the *B. subtilis* L-form, and/or the migration of dividing daughter plastids in opposite directions, as in the case of Mycoplasma, although the FtsZ-based division machinery is involved in the normal division process in land plants. In this regard, further studies are required.

Glaucophyte algae do not possess DRP5B and the chloroplasts have retained a thick PG layer between the two envelope membranes. This chloroplast division involves the inward growth of PG and probably also PG degradation at the division site, as in the case of bacterial cell division. Thus, the most plausible account is that DRP5B became integrated into the chloroplast division machinery in the common ancestor of red algae, green algae and land plants in parallel with the loss of the thick PG layer. DPR5B is also missing from certain eukaryotic genomes that possess chloroplasts or non-photosynthetic plastids. Other than the case of apicomplexans, in which another DRP is involved in plastid division, how chloroplasts are able to divide without DRP5B is still unclear. However, even in land plants, chloroplasts of DRP5B knockouts are still able to divide, although the efficiency of division is lower than in the wild type. In addition, the proplastid in the apical meristem apparently divides without DRP5B in the wild type. These facts raises the question whether DRP5B is indispensable for chloroplast division implying that DRP5B is rather a facilitator or a modulator of chloroplast division, or alternatively, that there is another as-yet unknown, redundant mechanism for chloroplast division. Thus, further studies using FtsZ or DRP5B knockouts or chloroplast-carrying eukaryotes that do not possess FtsZ or DRP5B will be needed to reveal the as-yet unknown mechanisms that are involved in chloroplast and non-photosynthetic plasyid division.

### Conflict of interest statement

The authors declare that the research was conducted in the absence of any commercial or financial relationships that could be construed as a potential conflict of interest.
